# Characterization of a Panel of Cross-Reactive Hantavirus Nucleocapsid Protein-Specific Monoclonal Antibodies

**DOI:** 10.3390/v15020532

**Published:** 2023-02-14

**Authors:** Aliona Avižinienė, Indrė Kučinskaitė-Kodzė, Rasa Petraitytė-Burneikienė, Aurelija Žvirblienė, Marc L. Mertens, Sabrina Schmidt, Mathias Schlegel, Erik Lattwein, Bernd Koellner, Rainer G. Ulrich

**Affiliations:** 1 Institute of Biotechnology, Life Sciences Center, Vilnius University, LT-10257 Vilnius, Lithuania; 2 Institute of Novel and Emerging Infectious Diseases, Friedrich-Loeffler-Institut (FLI), Federal Research Institute for Animal Health, 17493 Greifswald-Insel Riems, Germany; 3 German Society for Tissue Transplantation, Feodor-Lynen-Str. 21, 30625 Hannover, Germany; 4 Seramun Diagnostica GmbH, Spreenhagener Street 1, 15754 Heidesee, Germany; 5 Institute for Experimental Immunology, Euroimmun Medical laboratory Diagnostics AG, Seekamp 31, 23560 Luebeck, Germany; 6 Institute of Immunology, Friedrich-Loeffler-Institute, Federal Research Institute for Animal Health, 17493 Greifswald-Insel Riems, Germany; 7 German Centre for Infection Research, Partner Site Hamburg-Lübeck-Borstel-Riems, Südufer 10, 17493 Greifswald-Insel Riems, Germany

**Keywords:** monoclonal antibody, cross-reactivity, hantavirus, *Saccharomyces cerevisiae*, nucleocapsid protein, epitope mapping

## Abstract

Hantaviruses are emerging pathogens with a worldwide distribution that can cause life-threatening diseases in humans. Monoclonal antibodies (MAbs) against hantavirus nucleocapsid (N) proteins are important tools in virus diagnostics, epidemiological studies and basic research studies on virus replication and pathogenesis. Here, we extend the collection of previously generated MAbs raised against a segment of Puumala orthohantavirus (PUUV) N protein harbored on virus-like particles (VLPs) and MAbs against N proteins of Sin Nombre orthohantavirus/Andes orthohantavirus by generating nine novel MAbs against N proteins of Dobrava-Belgrade orthohantavirus (DOBV), Tula orthohantavirus (TULV), Thottapalayam thottimvirus (TPMV) and PUUV. In order to have a wide collection of well-described hantavirus-specific MAbs, the cross-reactivity of novel and previously generated MAbs was determined against N proteins of 15 rodent- and shrew-borne hantaviruses by different immunological methods. We found that all MAbs, excluding TPMV-specific MAbs, demonstrated different cross-reactivity patterns with N proteins of hantaviruses and recognized native viral antigens in infected mammalian cells. This well-characterized collection of cross-reactive hantavirus-specific MAbs has a potential application in various fields of hantavirus research, diagnostics and therapy.

## 1. Introduction

Hantaviruses are enveloped zoonotic viruses that belong to the family *Hantaviridae*, order *Bunyavirales*. Their genome consists of three single-stranded RNA segments (small, medium, large) of negative polarity that encode the nucleocapsid (N) protein, two surface glycoproteins (Gn, Gc) and RNA-dependent RNA polymerase, respectively [1,2]. The hantavirus N protein encapsidates viral genome segments forming ribonucleoprotein complexes [3]. Hantavirus N proteins are the most abundant structural proteins in virions and are detected in the cytoplasm of infected cells [4]. For these reasons, N proteins are frequently used as diagnostic antigens for serodiagnostics [5,6] including serotyping [7,8,9,10]. In addition, recombinant N proteins were applied for monoclonal antibody (MAb) production and subsequent application in basic research studies on hantavirus replication and pathogenesis [11,12,13,14,15,16].

A particular hantavirus is carried by one or few closely related reservoir species. These viruses are found in rodents (order Rodentia, families Muridae and Cricetidae), insectivorous bats (order Chiroptera), shrews (family Soricidae) and moles (family Talpidae, both order Eulipotyphla) [11,17,18,19]. In 2018, using a meta-transcriptomic approach, hantavirus-related genomic sequences were found in reptiles, ray-finned fish and jawless fish, showing that their prevalence is not fully investigated [20,21]. Moreover, soricid-, talpid- and chiropteran-borne hantaviruses are more genetically diverse than rodent-borne hantaviruses making evolutionary history of hantaviruses more complex than previously thought [18,22,23,24].

*Hantaan orthohantavirus* (HTNV), a prototype virus of the genus, and *Thailand orthohantavirus* (THAIV) are the best-known agents of Murinae-associated hantaviruses in Asia. Different genotypes of *Dobrava-Belgrade orthohantavirus* (DOBV) species are prevalent in Europe: Dobrava, Kurkino, Sochi and Saaremaa [25]. Differently to other hantaviruses, *Seoul orthohantavirus* (SEOV) is distributed worldwide due to wide dispersal of its rat reservoirs (*Rattus* spp.). *Puumala orthohantavirus* (PUUV) and *Tula orthohantavirus* (TULV) belong to the Arvicolinae-associated hantaviruses found in Europe, while *Prospect Hill orthohantavirus* (PHV), a virus non-pathogenic to humans, is spread in North America. Highly pathogenic Neotominae-associated *Sin Nombre orthohantavirus* (SNV) and Sigmodontinae-associated *Andes orthohantavirus* (ANDV) are found in North and South America, respectively [19,26,27]. *Thottapalayam thottimvirus* (TPMV) was initially thought to be an arbovirus, but was later identified to be a shrew-borne hantavirus occurring in South East Asia [28]. Phylogenetically divergent hantaviruses from multiple reservoir hosts may have a diverse range of sizes and morphologies of virions [29,30]. However, the existing differences at the amino acid sequence levels even between phylogenetically divergent rodent- and soricid-borne hantavirus N proteins do not alter the secondary structure of these proteins [31], maintaining the amino-terminal part as the immunodominant region bearing linear epitopes [4].

Hantaviruses cause an asymptomatic infection in their reservoir hosts. Humans can acquire infection from infected animals after the inhalation of aerosolized hantavirus-contaminated excreta such as urine, feces and saliva [32]. When transmitted to humans, the severity of the disease depends on the hantavirus species causing the disease. In Eurasia, hantaviruses are referred as Old World hantaviruses and can cause hemorrhagic fever with renal syndrome (HFRS) with case-fatality rate of up to 15% [19,33]. In Europe, the major causative agent of a mild-to-moderate form of HFRS (called nephropathia epidemica) is PUUV [33,34]. Hantaviruses detected in the Americas are referred as New World hantaviruses and include pathogens causing more lethal hantavirus cardiopulmonary syndrome (HCPS) having a case-fatality rate of up to 50% [35]. There is an increasing evidence of overlapping clinical symptoms of HFRS and HCPS; thus, the use of hantavirus disease is suggested as a single term for a complex global hantavirus infection [36,37].

The worldwide distribution of hantaviruses and the absence of approved vaccines against hantaviral infections in the United States and Europe [38,39] highlights the importance of having hantavirus-specific MAbs as therapeutical options for treating hantavirus disease [40,41] or for diagnostic applications [11,42,43,44].

Based on previous epitope-mapping studies, HFRS and HCPS patient antibodies as well as hantavirus-specific MAbs were shown to be mostly produced against the amino-terminal part of N proteins that is composed of linear epitopes [5,11,12,45,46]. Since hantavirus-specific MAbs can be raised against group-common, genus-common or serotype-specific epitopes, different cross-reactivity patterns of MAbs with N proteins of other hantaviruses have been reported [4,47,48]. While most of the MAbs raised against PUUV N protein were shown to be PUUV-specific [12], several MAbs against the amino-terminus of PUUV N protein cross-reacted with N proteins of hantaviruses from the New World, showing their antigenic similarities [47]. Additionally, MAbs raised against N protein of genetically divergent shrew-borne TPMV were shown to be TPMV-specific with almost no cross-reactions with rodent-borne hantaviruses [11].

Despite the provided research, there is a limited number of studies describing a wide collection of hantavirus-specific MAbs and comparing their cross-reactivities with N proteins of different hantaviruses. Our previously generated MAbs against N proteins of SNV/ANDV, as well as MAbs specific to the amino-terminal part of PUUV N protein, were shown to be cross-reactive and able to recognize native N proteins of different hantaviruses in infected cells [47,49]. In order to have a wider collection of well-characterized hantavirus-specific MAbs, we aimed to characterize the cross-reactivities of a panel of previously generated hantavirus-specific MAbs and novel MAbs against N proteins of rodent-borne DOBV, TULV, PUUV and shrew-borne TPMV against various hantavirus N and control proteins as well as test their applicability to recognize native viral proteins in infected mammalian cells.

## 2. Materials and Methods

### 2.1. Bioinformatics Analysis

The amino acid (aa) sequence identity (%) of hantavirus N proteins was evaluated using National Centre for Biotechnology Information (NCBI) protein–protein Basic Local Alignment Search Tool (BLAST) [50]. Multiple sequence alignment was generated using Clustal Omega software with default parameter settings [51]. For visualization, alignment results were imported into JalView Java alignment editor version 2.11.2.4 [52].

### 2.2. Construction of Plasmids

All DNA manipulations were performed according to the standard molecular biology protocols [53] and manufacturer recommendations. Enzymes and kits for DNA manipulation were purchased from Thermo Fisher Scientific Baltics (Vilnius, Lithuania).

For the construction of full-length N proteins and 120 amino acid-long truncated N proteins (N120) of DOBV strain Slovenia, TULV strain Moravia and TPMV, DNA sequences were PCR amplified using previously constructed plasmids pFX7_N-His_DOBV [54], pFX7_N-His_TULV [55], pFX7_N-His_TPMV [11] (Table 1) as templates and primers listed in Table S1. The obtained DNA fragments of TULV and TPMV were digested with XbaI restriction endonuclease (RE) and ligated into NheI-linearized bacterial expression plasmid pET28a^+^ (Merck, Darmstadt, Germany), while the DNA fragment of DOBV was digested with XhoI RE and inserted into pET28a^+^ (Merck). The constructed plasmids were screened in *Escherichia coli* DH5αF’ strain (Department of Eukaryote Gene Engineering, Institute of Biotechnology, Life Sciences Center, Vilnius University, Lithuania), verified by sequencing and used for transformation of *Escherichia coli* BL21 (DE3) cells (Novagene, Madison, WI, USA) (Table S2). The generation of pQE-derived plasmids (Qiagen, Hilden, Germany), encoding PUUV strain Vranica/Hällnäs N proteins spanning aa 1-433, 1-213 and 1-39+213-433 has been described previously [12,56].

For the production of N protein of *Tomato spotted wilt orthotospovirus* (TSWV; GenBank: D00645.1), the DNA fragment was amplified by PCR using plasmid pCS2_TSWV as a template [57] and primers listed in Table S1. The PCR products were cleaved with XbaI RE and inserted into XbaI-linearized yeast expression vector pFX7_N-His [54]. Constructed plasmid pFX7_N-His_TSWV was verified by sequencing and used for transformation of *Saccharomyces cerevisiae* yeast cells. The plasmids constructed in this study are listed in Table S2.

**Table 1:** List of His-tagged N proteins used for characterization of hantavirus-specific MAbs.

Origin of the N Protein			GenBank Accession No.	Differences of Published N Protein aa Sequences to Those Used Here ^a^	Reference
Virus Genus	Virus Species, Genotype, Strain	Abbreviation			
*Orthohantavirus*	*Dobrava-Belgrade virus*, Kurkino, strain Slovakia	DOBV-Slk	AY533118	-	[54]
*Orthohantavirus*	*Dobrava-Belgrade virus*, Dobrava, strain Slovenia	DOBV-Slo	L41916	-	[54]
*Orthohantavirus*	*Dobrava-Belgrade virus*, Saaremaa, strain Saar/90Aa/97	DOBV-Saa	AJ009775	R77K	[58]
*Orthohantavirus*	*Hantaan virus*, strain Fojnica	HTNV	M14626	G377D, A410V	[54]
*Orthohantavirus*	*Thailand virus*, strain Thai749	THAIV	AB186420	-	[59]
*Orthohantavirus*	*Seoul virus*, strain 80-39 *	SEOV	AY273791	-	[47,60]
*Orthohantavirus*	*Puumala virus*, strain Bavaria	PUUV-Bawa	JN696374	-	[61]
*Orthohantavirus*	*Puumala virus*, strain Kazan *	PUUV-Kaz	Z84204	S244C	[54]
*Orthohantavirus*	*Puumala virus*, strain Sotkamo *	PUUV-Sot	P27313	-	[54]
*Orthohantavirus*	*Puumala virus*, strain Vranica/Hällnäs	PUUV-Vra	U14137	-	[62]
*Orthohantavirus*	*Tula virus*, strain Moravia	TULV	Z69991	Y212H, R311A	[47,60]
*Orthohantavirus*	*Prospect Hill virus*, strain 3571	PHV	M34011	T7I, Q97R, E128G, H139Y	[47]
*Orthohantavirus*	*Sin Nombre virus*, strain 3H226	SNV	NC_005216	-	[47,63]
*Orthohantavirus*	*Andes virus*, strain AH-1	ANDV	AF004660	-	[47,64]
*Thottimvirus*	*Thottapalayam virus*	TPMV	AY526097	Q20K, A102T, G346S, G357A	[11]
*Phlebovirus*	*Rift valley fever virus*	RVFV	P21700	-	[65]
*Orthobunyavirus*	*Schmallenberg virus*	SBV	HE649914	-	[66]
*Tospovirus*	*Tomato spotted wilt virus*	TSWV	NC_002051	-	[57]

^a^ The first letter corresponds to the aa of N protein from the GenBank entry, the last letter corresponds to the aa used herein; the number refers to the position of aa residue within the N protein.* N proteins of *Dobrava-Belgrade orthohantavirus* strain Saaremaa, *Seoul orthohantavirus and Puumala orthohantavirus* strains Sotkamo and Kazan contain the entire aa sequences, whereas other hantavirus N proteins lack the amino-terminal methionine residue.

### 2.3. Synthesis and Purification of Recombinant N Proteins in S. cerevisiae and E. coli

*S. cerevisiae* Gcn2 strain (ATCC™4033642) was used for the synthesis of His-tagged full-length recombinant N proteins of hantaviruses, N proteins of *Rift valley fever phlebovirus* (RVFV) and TSWV. The N protein of *Schmallenberg orthobunyavirus* (SBV) was produced in *S. cerevisiae* AH22-214 strain (ATCC™ 38626). All N proteins used in this study are listed in Table 1.

Recombinant protein synthesis and purification by nickel-chelation chromatography under denaturing conditions were performed as described previously [54,62,66]. Briefly, pFX7_N-His-derived plasmids carrying the N protein-encoding sequences were transformed into *S. cerevisiae*. The yeast cells were grown with glucose-containing media with shaking at 30 °C for 20–24 h. After addition of induction media with galactose, the cells were grown for an additional 20–24 h. Collected yeast cells were suspended in phosphate disruption buffer (DB: 100 mM NaCl, 80 mM Na_2_HPO_4_, 24 mM NaH_2_PO_4_ (pH 7.5), 2 mM phenylmethylsulfonylfluorid (PMSF), 2 mM EDTA) and mechanically disrupted through vortexing with glass beads (0.5 mm diameter, Sigma Aldrich Co., St. Louis, MO, USA). The recombinant N proteins from the clarified yeast lysates were sedimented by centrifugation at 9400× *g* for 40 min. at 4 °C (Beckman Coulter Avanti J26 XP Centrifuge, Indianapolis, IN, USA). The protein pellets containing recombinant N proteins were dissolved in extraction buffer (DB buffer without PMSF and EDTA, supplemented with 1% Tween-20) and incubated on ice for 30 min. with gentle shaking. The proteins were sedimented by centrifugation at 9400× *g* for 40 min. at 4 °C (Beckman Coulter Avanti J26 XP Centrifuge, Indianapolis, IN, USA), resuspended in GuHCl buffer (100 mM NaH_2_PO_4_, 10 mM Tris-HCl, 6 M GuHCl, pH 8.0) and incubated for 12 h or several days at 4 °C. Recombinant protein purification was carried out according to Qiagen recommendations [67] with minor modifications as described by [54]. Collected protein fractions were dialyzed against phosphate buffer, lyophilized and stored at −20 °C.

For the epitope mapping of hantavirus-specific MAbs, the synthesis of full-length and 120-aa-long N proteins of DOBV-Slo, TULV and TPMV was carried out in *E. coli* strain BL21 (DE3) (Novagene). The plasmid-transformed *E. coli* cells were grown in LB medium at 37 °C until cell density reached 0.6–0.7 (OD_600_). Then, the recombinant protein synthesis was induced using different isopropyl-β-D-thiogalactoside (IPTG) (Thermo Fisher Scientific) concentrations and growing conditions. The synthesis of N and N120 proteins of TULV and TPMV was induced by adding 1 mM IPTG followed by an incubation for 3 h at 28 °C with shaking. The recombinant DOBV proteins were synthesized by adding 0.1 mM IPTG and incubating for 20 h at 24 °C with shaking. Then, the cells were collected by centrifugation at 1800× *g* for 10 min, resuspended in 1× protein dye buffer (0.05 M Tris-HCl (pH 6.8), 2% SDS (*w*/*v*), 0.05% bromophenol blue, 10% (*v*/*v*) glycerin, 5% 2-mercaptoethanol), heated for 10 min. at 100 °C and analyzed by sodium dodecyl sulfate polyacrylamide gel electrophoresis (SDS-PAGE). The pQE plasmid-driven production of recombinant PUUV strain Vranica/Hällnäs His-tagged N proteins (aa 1-433, aa 1-213 and aa 1-39+213-433) and the didydrofolate reductase control protein (DHFR) was performed in *E. coli* M15pREP4 cells as previously described [12]. The affinity chromatography purification followed a published standard protocol based on the recommendations of the ma-nufacturer of the pQE expression system [67].

### 2.4. Production and Purification of Monoclonal Antibodies

BALB/c mice were immunized with amino-terminally His-tagged full-length N proteins of PUUV strain Bavaria (PUUV-Bawa), TULV strain Moravia, DOBV genotype Dobrava, strain Slovenia (DOBV-Slo) and TPMV (permit no. 28/17; Landesamt für Landwirtschaft, Lebensmittelsicherheit und Fischerei, Rostock, Germany).

A panel of hybridomas, generated by fusion of spleen cells from immunized mice and cultured myeloma cells, was found to produce MAbs specifically reactive with hantavirus N proteins in indirect ELISA and Western blot tests. MAbs-producing hybridomas were cloned by limiting dilution in hybridoma growing medium (Ham’s F-12 medium with Glutamine, Iscove’s medium containing 25 mM HEPES and L-Glutamine (PAA Cell Culture Company, Cambridge, UK) mixed 1:1 with 10% fetal calf serum (Biochrom, Cambridge, UK)) in Corning^®^ tissue culture-treated 96-well plates (Merck) at 37 °C and 5% CO_2_. All MAbs were purified from hybridoma growth medium by affinity chromatography on protein A sepharose (rProtein A Sepharose Fast Flow, GE Healthcare, Chicago, IL, USA) according to manufacturer’s recommendations. Heavy- and light-chain types of MAbs were determined by Mouse Immunoglobulin Isotyping ELISA Kit (ISO-2, Sigma Aldrich, Darmstadt, Germany).

In this study, two panels of hantavirus-specific MAbs were used—novel and previously generated. Nine novel MAbs are raised against full-length N proteins of DOBV-Slo (MAbs DOBV 2, 3-2, 4 and F2-2), TULV (MAb TULV 1), PUUV-Bawa (MAbs PUUV 1 and 10-3) and TPMV (MAbs TPMV 6 and 9-1). Previously generated murine MAbs were raised against a segment of PUUV-Vra N protein presented on hamster polyomavirus-VP1-derived VLPs (MAbs 2C6, 5C5, 5E11 and 7A5) [49] as well as against full-length SNV/ANDV N proteins (MAbs 4H3, 7G2) [47].

### 2.5. Indirect ELISA and Determination of the Apparent Dissociation Constants (K_d_)

The cross-reactivity of MAbs was evaluated by indirect ELISA as previously described by [33]. Briefly, polystyrene microtiter plates (Nerbe plus GmbH, Winsen/Luhe, Germany) were coated with hantavirus N proteins and control proteins of SBV, RVFV and TSWV by adding 50 µL of the antigen solution (5 µg/mL) in coating buffer (50 mM Na-carbonate, pH 9.5) and incubated at 4 °C overnight. The plates were blocked for 1 h at room temperature (RT) with 200 µL 2% bovine serum albumin (BSA) diluted in phosphate-buffered saline (PBS: 137 mM NaCl, 27 mM KCl, 8 mM Na_2_HPO_4_ × 12 H_2_O, 1.4 mM KH_2_PO_4_). Each purified MAb was diluted in 1% BSA in PBS-T (PBS containing 0.1% Tween-20) in 3-fold steps starting from 10 µg/mL concentration and incubated (100 µL/ well) for 1 h at RT. As controls for an indirect ELISA, anti-SBV MAb [66], anti-RVFV MAb [68] and anti-TSWV rabbit polyclonal antibodies [69] were used. After washing the plates five times with PBS-T, horseradish peroxidase (HRP)-labelled goat anti-mouse IgG (BioRad, Hercules, CA, USA) were diluted 1:5000 in 1% BSA in PBS-T, and 100 µL was added to each well. For negative control reactions, HRP-labelled goat anti-rabbit IgG antibody (BioRad) was added to wells with anti-TSWV rabbit polyclonal antibodies using the same dilution as described. Plates were incubated for 1 h at RT and then washed five times with PBS-T. The antibody binding was visualized by the addition of tetramethylbenzidine (TMB) substrate (Clinical Science Products, Mansfield, MA, USA). The reaction was stopped by adding 1 M H_2_SO_4_. The optical density (OD) values were measured at 450 nm in microtiter plate by MultiSkan^TM^ GO Microplate reader (Thermo Fisher Scientific, Waltham, MA, USA).

The apparent dissociation constants (K_d_) of the MAbs and N proteins were calculated from titration curves obtained by incubating plate-coated N proteins with decreasing amounts of MAbs ranging from 3 × 10^−8^ M to 1 × 10^−13^ M. The K_d_ for each MAb was described as the molar concentration (M) of MAb that gives half of the maximum OD_450_ value.

### 2.6. SDS-PAGE and Western Blot Analysis

The protein samples were mixed with 2× protein dye buffer (0.1 M Tris-HCl (pH 6.8), 4% SDS (*w*/*v*), 0.1% bromophenol blue, 20% (*v*/*v*) glycerin, 10% 2-mercaptoethanol), heated for 10 min. at 100 °C and fractionated by SDS-PAGE in 12% gels under reducing conditions. The gels were stained with Coomassie brilliant blue (Sigma Aldrich) for protein band visualization or transferred onto a polyvinylidene difluoride (PVDF) membrane (Carl Roth GmbH and Co., Karlsruhe, Germany) for Western blot analysis under semi-dry conditions, as previously described [70]. Briefly, after protein transfer from the polyacrylamide gels onto PVDF membranes, the membranes were blocked with 1× Roti-Block solution (Carl Roth GmbH and Co.) for 1 h. Then, the membranes were rinsed with TBS-T (20 mM Tris, 137 mM NaCl (pH 7.6), 0.1% Tween-20) and incubated with MAbs (1 µg/mL in TBS-T) overnight at RT. As controls for Western blot analysis, anti-SBV hybridoma cell culture supernatant (1:10 in TBS-T) [66], anti-RVFV MAb [68] and anti-TSWV rabbit polyclonal antibodies [69] were used. After membrane rinsing with TBS-T, goat HRP-labelled anti-mouse IgG (BioRad) or goat HRP-labelled anti-rabbit IgG (BioRad) were diluted 1:3000 in TBS-T followed by an incubation for 2 h at RT. After membrane rinsing with TBS-T and then with TBS (TBS-T buffer without 0.1% Tween-20), the enzymatic reaction was visualized by adding 4-chloro-1-naphthol and hydrogen peroxide (Arcos Organics, Geel, Belgium) in TBS.

For the epitope mapping of PUUV 1 and PUUV 10-3 MAbs in Western blot assay, a slightly different methodology was used. Briefly, protein samples of PUUV-Vra N proteins (aa 1-213, aa 1-39+213-433 and the full-length N protein aa 1-433 as well as the DHFR control protein) were transferred onto PVDF membranes. The membranes were blocked with 5% nonfat dry milk (Hobbybäcker-Versand, Bellenberg, Germany) in PBS-T overnight at RT. Then, the membranes were subjected with primary anti-His MAb (1:2500 in PBS-T with 5% nonfat dry milk) (Novagen, Darmstadt, Germany), PUUV 1 or PUUV 10-3 cell culture supernatants (1:2 in PBS-T with 5% nonfat dry milk) followed by incubations with HRP-labeled goat anti-mouse IgG (H + L) (BioRad), diluted 1:3000 in PBS-T, as secondary antibodies. Chemiluminescent signal was developed using Clarity™ Western ECL Substrate (BioRad, Feldkirchen, Germany) at a 60 sec. exposure time at the VersaDoc imaging system (BioRad).

### 2.7. Immunofluorescence Assay (IFA)

IFA analysis was performed using “*IIFT: Hantavirus Mosaic Global*” kit (Euroimmun, Luebeck, Germany). The kit contained microscope slides with ten reaction fields each containing eight biochips with HTNV-, PUUV-Kaz-, SEOV-, DOBV-Saa-, DOBV-Slo-, SNV- and ANDV- infected and non-infected Vero cells. The reactivity of hantavirus-specific MAbs was tested according to the manufacturer’s recommendations. Briefly, MAbs were diluted to 2 µg/mL in sample buffer provided in the kit. As control for IFA, earlier described ANDV/SNV-(4H3, 7G2) and PUUV-specific MAbs (2C6, 5C5, 5E11 and 7A5) were used [47,49]. A serum pool from PUUV-, SEOV- and SNV-infected patients and sera from a healthy blood donor (provided in the kit) were used as positive and negative controls, respectively. The MAb and serum sample dilutions were placed onto the microscopic slides with hantavirus-infected cells and incubated for 2 h at RT. Then, the slides were rinsed three times by immersing them in PBS for 5 min. Fluorescein isothiocyanate (FITC)-conjugated goat anti-mouse immunoglobulin G (IgG) (H + L) (Thermo Fisher Scientific) for MAbs and FITC-conjugated anti-human IgG ready-to-use for human serum control samples (provided in the kit) were used as secondary antibodies. Following a 1 h incubation in the dark at RT, the slides were rinsed as described above, embedded with the mounting medium, cover slipped and evaluated by the fluorescence microscope (Nikon Eclipse Ti, Nikon, Japan) using “NIS-Elements Basic research“ software. Positive reactions were characterized by a granular intensity of the immunofluorescence signal in the cytoplasm of hantavirus-infected cells.

## 3. Results

### 3.1. Bioinformatics Analysis of Hantavirus N Proteins

**Figure 1:**
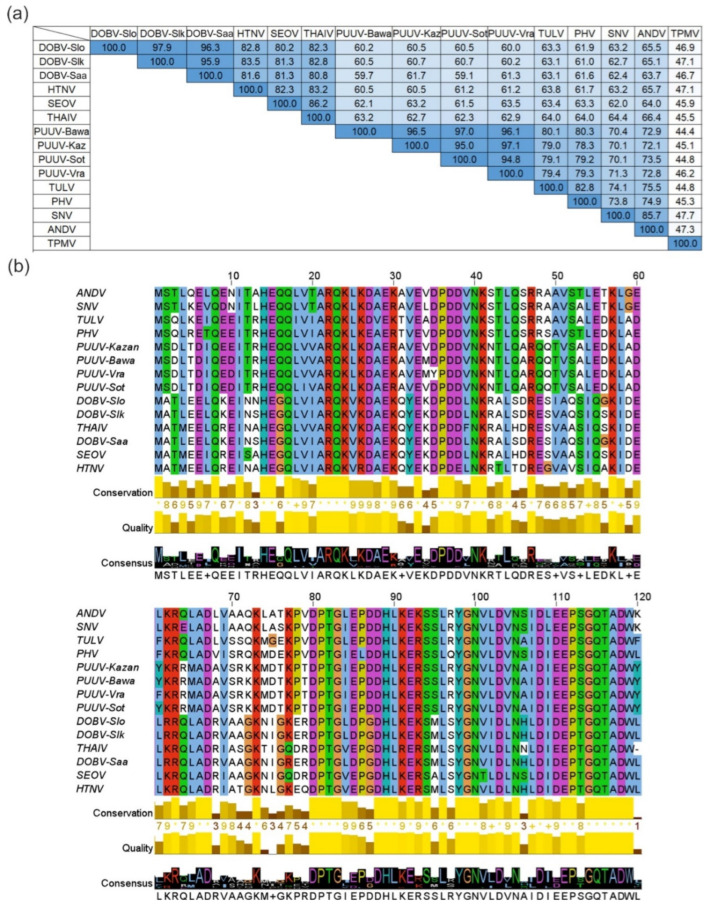
Pairwise identity values and multiple N protein aa sequence alignment of hantaviruses. (**a**) Pairwise identity values (%) of hantavirus N protein aa sequences. Color intensity indicates the aa identity level between N protein sequences. (**b**) Multiple sequence alignment of hantavirus N proteins. Each residue in the alignment is assigned to a color scheme used for alignments in ClustalX if the aa profile of the alignment at that position meets criteria specific for the residue type (blue color—hydrophobic residues, red—positive charge, magenta—negative charge, green—polar, orange—glycines, yellow—prolines, cyan—aromatic, white—unconserved if none of the criteria are met). The bar diagrams and the numbers under the alignment indicate sequence conservation level, alignment quality and the consensus sequence. *Dobrava-Belgrade orthohantavirus* genotype Dobrava, strain Slovenia (DOBV-Slo), genotype Kurkino, strain Slovakia (DOBV-Slk) and genotype Saaremaa (DOBV-Saa); *Hantaan orthohantavirus* (HTNV); *Seoul orthohantavirus* (SEOV); *Thailand orthohantavirus* (THAIV); *Puumala orthohantavirus* strains Bavaria (PUUV-Bawa), Kazan (PUUV-Kaz), Sotkamo (PUUV-Sot), Vranica/Hällnäs (PUUV-Vra); *Tula orthohantavirus* (TULV); *Prospect Hill orthohantavirus* (PHV); *Sin Nombre orthohantavirus* (SNV); *Andes orthohantavirus* (ANDV); *Thottapalayam thottimvirus* (TPMV). The aa sequence identity levels were evaluated using NCBI protein–protein BLAST [50], multiple aa sequence alignment was assembled with Clustal Omega [51] and visualized with JalView software [52].

Based on the results of the bioinformatics analysis, rodent-borne hantavirus N proteins have high aa sequence identity values varying from 59.1% to 97.9% (Figure 1). The highest sequence identity values varying from 95.9% to 97.9% were determined for DOBV strains Slovenia (DOBV-Slo), Slovakia (DOBV-Slk) and Saaremaa (DOBV-Saa). Four PUUV strains, Bavaria (PUUV-Bawa), Kazan (PUUV-Kaz), Sotkamo (PUUV-Sot) and Vranica/Hällnäs (PUUV-Vra), form another group of viruses sharing high sequence identity values from 94.8% to 97.1%. In addition, N proteins of HTNV, SEOV and THAIV share sequence identity values from 82.3% to 86.2%. Our data further revealed that the N proteins of SNV and ANDV as well as TULV and PHV share high sequence identity values of 85.7% and 82.8%, respectively. As shown in Figure 1a, shrew-borne TPMV is the most divergent hantavirus used in this study—its N protein shares comparatively low aa sequence identity with other hantavirus N proteins varying from 44.4% to 47.7%.

### 3.2. Generation, Initial Characterization and Epitope Mapping of DOBV, TULV, TPMV and PUUV N Protein-Specific MAbs

BALB/c mice were immunized with *S. cerevisiae*-produced recombinant N proteins of PUUV, TULV, DOBV and TPMV and a panel of hybridomas stably producing hantavirus N protein-specific MAbs were generated. After characterization by ELISA and Western blot test, nine hybridomas were selected for cloning procedures and subsequently used for more detailed investigations including cross-reactivity study by indirect ELISA, Western blot test and IFA. Seven MAbs (DOBV 2, DOBV 3-2, DOBV 4, PUUV 1, PUUV 10-3, TPMV 6 and TPMV 9-1) were of IgG1 subtype and two MAbs (DOBV F2-2 and TULV 1) were of IgG2a subtype.

**Figure 2:**
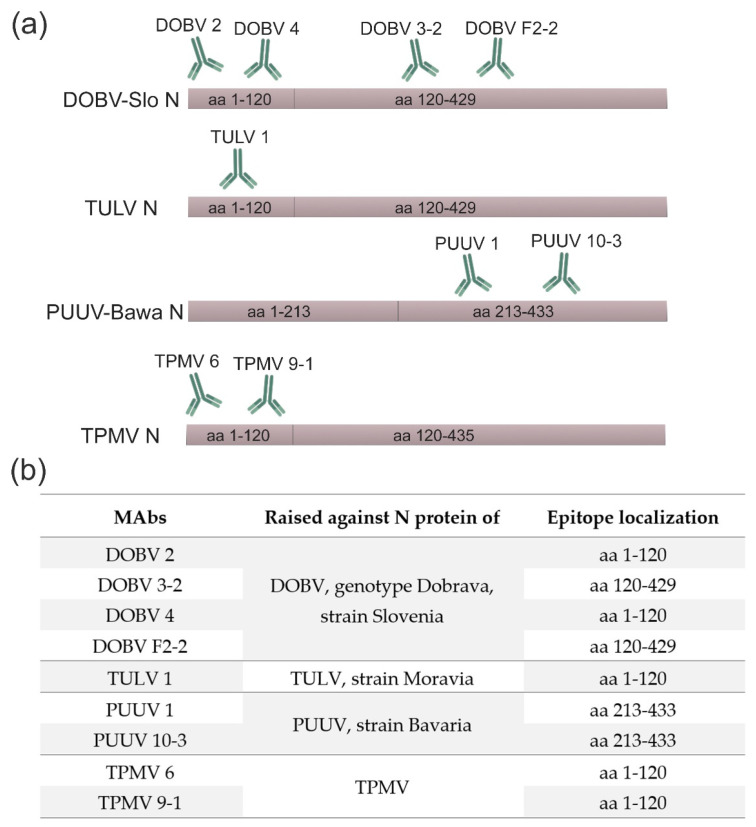
Epitope mapping of MAbs raised against N proteins of DOBV, TULV, PUUV and TPMV. (**a**) A schematic representation of binding regions of hantavirus-specific MAbs within corresponding hantavirus N proteins. (**b**) Epitope mapping data. DOBV-Slo—*Dobrava-Belgrade orthohantavirus* genotype Dobrava, strain Slovenia; TULV—*Tula orthohantavirus*; PUUV-Bawa—*Puumala orthohantavirus* strain Bavaria; TPMV—*Thottapalayam thottimvirus*.

For the epitope mapping of DOBV-, TULV-, TPMV- and PUUV-specific MAbs, *E. coli*-produced His-tagged full-length N proteins and truncated proteins (DOBV, TULV and TPMV: N120 and PUUV: N1-213 and N1-39+213-433) of these hantaviruses were used as antigens. A Western blot analysis of bacterial cell lysates with produced recombinant N proteins revealed that the epitopes recognized by the majority of hantavirus-specific MAbs were located in the amino-terminal immunodominant region within aa residues 1-120. Due to lacking reactivity of DOBV 3-2 and DOBV F2-2 MAbs with the corresponding truncated proteins, the epitopes of these MAbs may be located within 120–429 aa region of N proteins (Figure 2 and Figure S1). The MAbs PUUV 1 and 10-3 did not react with truncated protein N1-213, but showed reactivity with the fusion protein (aa 1-39+213-433) suggesting an epitope localization in the carboxy-terminal half of the N protein (aa 213-433) (Figure 2 and Figure S2).

### 3.3. Cross-Reactivity Evaluation of Hantavirus-Specific MAbs with Hantavirus N Proteins in ELISA and Western Blot Assay

The cross-reactivity pattern of hantavirus-specific MAbs was evaluated in parallel by indirect ELISA and Western blot assay using yeast-produced full-length N proteins of 14 rodent-borne hantaviruses and the shrew-borne TPMV. For ELISA, the affinity between MAbs and the N proteins of different hantaviruses was evaluated by calculating the apparent dissociation constants (K_d_). According to the ELISA and Western blot assay, DOBV- and TULV-specific MAbs showed the broadest cross-reactivities and recognized almost all N proteins of rodent-borne hantaviruses used in this study (Table 2 and Table 3, Figure S3). All DOBV-specific MAbs recognized N proteins of DOBV genotypes Dobrava, Kurkino and Saaremaa and HTNV, SEOV and THAIV. In the ELISA, all DOBV-specific MAbs recognized SNV N protein, while DOBV 3-2 and DOBV F2-2 MAbs did not show reactivity with this protein in the Western blot assay. Additionally, all DOBV-specific MAbs recognized ANDV N protein in the Western blot assay, but the reaction of DOBV 4 MAb with ANDV N was not observed in the ELISA. From all DOBV-specific MAbs, only the DOBV 2 MAb recognized N protein of PHV and three PUUV strains (Bavaria, Kazan, Sotkamo) in the ELISA and Western blot assay. The DOBV 3-2 MAb recognized PUUV-Bawa, but not N proteins of other PUUV strains. Compared to other DOBV-specific MAbs, only the DOBV 4 MAb recognized TULV N protein in both assays. Similar to DOBV-specific MAbs, TULV-specific MAb TULV 1 showed a broad cross-reactivity with other hantavirus N proteins. In the ELISAand Western blot assay, the TULV 1 MAb recognized all N proteins of rodent-borne hantaviruses used in this study, except the N protein of PUUV-Vra (Table 2 and Table 3, Figure S3).

Compared to DOBV- and TULV-specific MAbs, PUUV-specific MAbs (PUUV 1 and PUUV 10-3) were less cross-reactive. In the ELISA and Western blot assay, PUUV 1 and PUUV 10-3 MAbs recognized all N proteins of PUUV strains used in this study—Bavaria, Kazan, Sotkamo, Vranica/Hällnäs. Additionally, PUUV 10-3 MAb recognized N proteins of ANDV and PHV in the ELISA. Compared with DOBV-, TULV- and PUUV-specific MAbs, TPMV-specific MAbs (TPMV 6 and TPMV 9-1) exclusively reacted with the TPMV N protein and did not show cross-reactivity with N proteins of rodent-borne hantaviruses (Table 2 and Table 3, Figure S3).

The cross-reactivities of previously generated MAbs raised against amino-terminal region of PUUV-Vra N protein presented on polyomavirus-derived VLPs (MAbs 2C6, 5C5, 5E11 and 7A5) as well as MAbs against full-length SNV/ANDV N proteins (4H3, 7G2) [47,49] were additionally evaluated with N proteins of DOBV genotypes Kurkino and Saaremaa, THAIV, PUUV strain Bavaria and TPMV in the ELISA and Western blot assay. The data almost completely correspond to the previously published results with a few exceptions (see Table 2 and Table 3). Additionally, none of the previously generated MAbs recognized TPMV N protein in the ELISA and Western blot assay. The specificity of the ELISA and Western blot assay was confirmed by using N proteins of SBV, RVFV and TSWV as negative controls that did not show any reactivity with hantavirus-specific MAbs (Table 2 and Table 3).

### 3.4. Reactivities of Hantavirus-Specific MAbs with Hantavirus-Infected Vero Cells in IFA

The immunoreactivity of DOBV-, TULV- and PUUV-specific MAbs was additionally analyzed with hantavirus-infected cells (Table 4). TPMV-specific MAbs were excluded from the immunofluorescence investigation since they reacted in the ELISA and Western blot assay exclusively with the TPMV N protein.

DOBV-, TULV- and PUUV-specific MAbs recognized at least one native viral protein found in hantavirus-infected cells and the reactivity pattern correlated with the results of the ELISA and Western blot assay. The TULV 1 MAb showed the broadest cross-reactivity in IFA and reacted with DOBV-Slo-, DOBV-Saa-, HTNV-, SEOV-, PUUV-Kaz- and SNV-infected cells. The DOBV 2 MAb confirmed its previously reported cross-reactivity in the ELISA and the Western blot assay, except there was no reactivity with the PUUV-Kaz antigen. The immunoreactivity of DOBV 3-2, DOBV 4 and DOBV F2-2 MAbs slightly differed from that observed earlier in the ELISA and Western blot assay: the DOBV 4 MAb additionally reacted with ANDV, while DOBV 3-2 and DOBV F2-2 MAbs did not recognize SNV antigens. In IFA, PUUV 1 and PUUV 10-3 MAbs exclusively recognized PUUV-Kaz-infected cells and did not show cross-reactivity with other hantaviruses (Table 4).

Previously generated MAbs, raised against a segment of the PUUV-Vra N protein (aa 1-120) and against the entire SNV/ANDV N proteins, were used for the evaluation of the test’s repeatability [47,49]. In general, all six MAbs demonstrated similar results as published before, with few exceptions (Table 4). The fluorescence signal between 7G2 MAb with HTNV-infected cells was stronger than previously reported, also it recognized ANDV antigens. PUUV-Vra N protein segment-specific MAbs, except 2C6 MAb, showed a broader cross-reactivity pattern: the fluorescence signal of 5C5 MAb with SNV and ANDV antigens was stronger, but there was no reaction with SEOV antigen. While the fluorescence signal of 5E11 MAb with DOBV-Slo, DOBV-Saa and ANDV was more intensive than previously reported, the reactivity with SEOV antigens was weaker. Finally, the 7A5 MAb additionally reacted with ANDV, but it was weaker in its recognition of PUUV-Kaz antigen (Table 4).

## 4. Discussion

Previously generated MAbs against yeast-produced N proteins of SNV/ANDV [47] and the segment of N protein of PUUV-Vra [49] had different cross-reactivity patterns with hantavirus N proteins and were able to detect native viral antigens in virus-infected cells. The existing collection of MAbs was improved by generating nine novel MAbs raised against N proteins of rodent-borne DOBV, TULV, PUUV- and shrew-borne TPMV. The immunological investigation of novel and previously generated MAbs using N proteins of 15 hantaviruses, including four PUUV strains and three DOBV genotypes, resulted in the identification of different cross-reactivity patterns in the ELISA, Western blot assay and IFA. The additional use of N proteins of other bunyaviruses, namely SBV, RVFV and TSWV, confirmed the hantavirus specificity of the MAbs.

The N proteins of rodent-borne hantaviruses used in this study share high aa sequence identity values varying from 59.1% to 97.9%. DOBV- and TULV-specific MAbs showed the broadest cross-reactivity with almost all N proteins of rodent-borne hantaviruses in the ELISA, Western blot assay and IFA. The most cross-reactive TULV 1 MAb recognized almost all rodent-borne hantavirus N proteins, except the N protein of PUUV-Vra in the ELISA and Western blot assay. From four DOBV-specific MAbs, only DOBV 2 MAb recognized N proteins of PUUV strains with the exception of PUUV-Vra. The non-reactivity of hantavirus-specific MAbs with N protein of PUUV-Vra could in part be explained by five aa exchanges within the N protein of PUUV-Vra strain (D35Y, Y61F, I157Y, E234D and E429D), compared to the N proteins of PUUV strains Bavaria, Sotkamo and Kazan. Interestingly, for MAb TULV1 the aa exchanges at positions 35 and 61 might be important for antigen recognition, as the epitope region was mapped to aa 1-120 of N protein. It has been discussed earlier that the aa substitution of the N protein of PUUV Vranica/Hällnäs at position 35, where the aspartic acid residue (D) is present in all hantavirus N proteins is changed to a tyrosine residue (Y) (D35Y), is located in a highly conserved immunodominant region and can be responsible for the different reactivity of hantavirus-specific MAbs with PUUV strains [12,47,61,71]. Moreover, MAb 2C6 raised against the amino-terminal region of PUUV-Vra N protein exclusively reacted with the homologous N protein, but not with the N proteins of any other PUUV strain (this study and [49]). This amino acid exchange at position 35 was also found to influence the protective immune response induced in the bank vole model [72].

While novel PUUV-specific MAbs mainly recognized N proteins of PUUV strains used in this study (Bavaria, Kazan, Sotkamo and Vranica/Hällnäs), PUUV 10-3 MAb was more cross-reactive and additionally recognized N proteins of PHV and ANDV in the ELISA. A higher level of cross-reactivity was also observed for previously generated MAbs 5C5, 5E11 and 7A5 raised against truncated PUUV-Vra N protein presented on VLPs (this study and [47]). In general, lower cross-reactivity of PUUV-specific MAbs with N proteins of other rodent-borne hantaviruses could be explained by the divergence of PUUV N protein, and that of the strain Vranica/Hällnäs in particular [73]. In line with previous data [61], the different reactivity of hantavirus-specific MAbs with N proteins of PUUV strains indicates higher antigenic similarities between Bavaria, Kazan and Sotkamo strains, but not with PUUV strain Vranica/Hällnäs.

In contrast to the highly cross-reactive MAbs of rodent-borne hantaviruses, a different reactivity pattern was observed with novel soricomorph-borne TPMV-specific MAbs. MAbs TPMV 6 and TPMV 9-1 were TPMV N protein-specific and did not show cross-reactivity with N proteins of rodent-borne orthohantaviruses. Our data confirm previously reported results for other TPMV-specific MAbs [11], concluding that shrew-borne TPMV is more diverse than rodent-borne hantaviruses causing the lack of cross-reactivity with other hantaviral N proteins [18,74].

PUUV-, TULV- and DOBV-specific MAbs recognized denatured, SDS-treated proteins in the ELISA and Western blot assay, as well as native viral antigens in hantavirus-infected cells. Reported reactivities let us hypothesize that novel MAbs may target linear epitopes common in denatured yeast-produced N proteins and native viral antigens in hantavirus-infected cells confirming the antigenic similarity between yeast-produced and native viral N proteins. In line with this assumption, previously generated hantavirus-specific MAbs also were found to recognize linear epitopes within N proteins [47,49,75,76,77], except several PUUV-specific MAbs recognizing conformation-dependent epitopes [12].

In the second part of this study, the cross-reactivity of previously generated SNV/ANDV N protein-specific MAbs, as well as MAbs specific to PUUV-Vra N protein segment harbored on hamster polyomavirus VLPs [47,49], was re-evaluated adding additional N proteins of DOBV-Slk, DOBV-Saa, THAIV, PUUV-Bawa, PHV and TPMV. Here, we observed a good correlation with previous results and demonstrated that cross-reactive MAbs 7G2, 5C5, 5E11 and 7A5 additionally recognized N proteins of rodent-borne hantaviruses, while highly specific MAbs 4H3 and 2C6 did not show reactivity with any of the additional hantavirus N proteins. Negative control antigens of bunyaviruses, namely RVFV, SBV and TSWV, were not recognized by any of the novel hantavirus-specific MAbs and previously generated MAbs showing the high specificity of performed immunological tests.

The reactivities of hantavirus-specific MAbs with denatured and native N proteins in the Western blot analysis and IFA indicate the presence of linear epitopes. Based on the results of epitope mapping studies, novel MAbs against N proteins of DOBV, TULV and TPMV were mostly produced against the amino-terminal immunodominant region (aa 1-120) of the corresponding N proteins. This is in line with previous investigations that the amino-terminus represents an immunodominant region of hantavirus N proteins and most of the MAbs recognize this region [11,12,78]. In this study, only MAbs PUUV 1, PUUV 10-3, DOBV 3-2 and DOBV F2-2 did not react with the amino-terminal region of the hantaviral proteins indicating their likely epitope regions located within aa 213-433 and aa 120-429 of N proteins, respectively.

Taken together, the broad cross-reactivity of novel DOBV- and TULV-specific MAbs and previously characterized MAbs 7G2, 5C5 and 5E11 with N proteins of other hantaviruses confirms the presence of highly cross-reactive, conserved epitopes within amino-terminal immunodominant region of hantaviral N proteins [12,47]. In line with these data, the majority of rodent-borne hantavirus-specific MAbs is cross-reactive and can recognize different hantavirus N proteins, while shrew-borne TPMV-specific MAbs do not show cross-reactivity with other hantavirus N proteins [11].

## 5. Conclusions

The existing collection of hantavirus-specific MAbs raised against a segment of PUUV N protein (2C6, 5C5, 5E11, 7A5; [49]), MAbs raised against N proteins of SNV/ANDV (4H3, 7G2; [47]) and MAbs raised against TPMV N protein [11] was extended by generating nine novel DOBV, TULV, TPMV and PUUV N protein-specific MAbs. An immunological analysis revealed their different cross-reactivity pattern with 15 full-length hantavirus N proteins. Due to the high aa sequence similarity of rodent-borne hantavirus N proteins, DOBV- and TULV-specific MAbs showed the broadest cross-reactivity with other hantavirus N proteins, while TPMV-specific MAbs did not show cross-reactivity with rodent-borne hantaviruses and reacted exclusively with the shrew-borne TPMV N protein. We showed that hantavirus-specific MAbs recognized denatured hantavirus N proteins in the ELISA and Western blot assay as well as native viral antigens in infected mammalian cells. This well-characterized collection of hantavirus-specific MAbs represents a valuable tool for diagnostic and basic research applications, while TPMV-specific MAbs might be used for the detection of TPMV infections in humans or animals. In particular, the highly cross-reactive MAb TULV 1 might be used for broad antigen screening of rodent reservoir tissues not only to detect known rodent-borne hantaviruses but also novel ones. In addition, this and other MAbs characterized here might be used for the development of rapid diagnostic assays or competitive ELISAs and should be evaluated for their antiviral activity in vivo.

## Figures and Tables

**Table 2:** Reactivities of hantavirus-specific MAbs with full-length hantavirus N proteins and control proteins in ELISA.

Recombinant N Proteins	Reactivity of Hantavirus-Specific MAbs														
	Novel MAbs									Previously Generated MAbs					
	DOBV 2	DOBV 3-2	DOBV 4	DOBV F2-2	TULV 1	PUUV 1	PUUV 10-3	TPMV 6	TPMV 9-1	Against N Proteins of SNV/ANDV		Against PUUV-Vra N Protein Segment (aa 1-120)			
										4H3 ^a^	7G2 ^a^	2C6 ^b^	5C5 ^b^	5E11 ^b^	7A5 ^b^
DOBV-Slo	+	++	+++	+++	+	−	−	−	−	− *	− *	− *	− *	++ *	− *
DOBV-Slk	++	++	+++	+++	+	−	−	−	−	−	−	−	−	++	−
DOBV-Saa	++	+++	+++	+++	+	−	−	−	−	−	−	−	−	++	−
HTNV	++	+++	+++	+++	+++	−	−	−	−	− *	+++ (+) *	− *	− *	+ (−) *	− *
SEOV	+	+++	+++	+++	+++	−	−	−	−	− *	+++ *	− *	+ (−) *	+++ *	− *
THAIV	+++	+++	+	+++	+	−	−	−	−	−	+	−	−	+	−
PUUV-Bawa	++	+	−	−	+++	+++	+++	−	−	−	+	−	+	+++	+++
PUUV-Kaz	++	−	−	−	+	+++	+++	−	−	− *	+ *	− *	+ *	+++ *	+++ *
PUUV-Sot	+++	−	−	−	+	+++	+++	−	−	− *	++ *	− *	+ *	+++ *	+++ *
PUUV-Vra	−	−	−	−	−	+++	+++	−	−	− *	++ *	+++ *	+++ *	+++ *	+++ *
TULV	−	−	+	−	+++	−	−	−	−	− *	++ *	− *	+ *	+++ *	++ *
PHV	++	−	−	−	+++	−	+	−	−	−	+++	−	+	++	−
SNV	+++	+	+	+	+++	−	−	−	−	+++ *	+++ *	− *	++ *	++ *	+++ *
ANDV	++	+++	−	+++	+++	−	++	−	−	+++ *	+++ *	− *	+ *	++ *	+++ *
TPMV	−	−	−	−	−	−	−	+++	+++	−	−	−	−	−	−
SBV	−	−	−	−	−	−	−	−	−	−	−	−	−	−	−
RVFV	−	−	−	−	−	−	−	−	−	−	−	−	−	−	−
TSWV	−	−	−	−	−	−	−	−	−	−	−	−	−	−	−

*Dobrava-Belgrade orthohantavirus,* genotype Dobrava, strain Slovenia (DOBV-Slo), genotype Kurkino, strain Slovakia (DOBV-Slk); genotype Saaremaa (DOBV-Saa); *Hantaan orthohantavirus* (HTNV); *Seoul orthohantavirus* (SEOV); *Thailand orthohantavirus* (THAIV); *Puumala orthohantavirus* strains Bavaria (PUUV-Bawa), Kazan (PUUV-Kaz), Sotkamo (PUUV-Sot), Vranica/Hällnäs (PUUV-Vra); *Tula orthohantavirus* (TULV); *Prospect Hill orthohantavirus* (PHV); *Sin Nombre orthohantavirus* (SNV); *Andes orthohantavirus* (ANDV); *Thottapalayam thottimvirus* (TPMV); *Schmallenberg orthobunyavirus* (SBV); *Rift valley fever phlebovirus* (RVFV)*; Tomato spotted wilt tospovirus* (TSWV). Apparent K_d_ values determined by an indirect ELISA are indicated: − (no reactivity), K_d_ > 1 × 10^−8^ M; + (weakly positive reactivity), K_d_ = 1 × 10^−8^–1 × 10^−9^ M; ++ (positive reactivity), K_d_ = 1 × 10^−9^–1 × 10^−10^ M; +++ (strongly positive reactivity), K_d_ < 1 × 10^−10^ M. ^a^, ^b^—MAbs described in [47,49], respectively. *—previously determined reactivity of MAbs in [47]. When current reactivity does not confirm previous results, it is indicated before the bracket.

**Table 3:** Reactivity patterns of hantavirus-specific MAbs with hantavirus N proteins and control proteins in Western blot assay.

Recombinant N Proteins	Reactivity of Hantavirus-Specific MAbs														
	Novel MAbs									Previously Generated MAbs					
	DOBV 2	DOBV 3-2	DOBV 4	DOBV F2-2	TULV 1	PUUV 1	PUUV 10-3	TPMV 6	TPMV 9-1	Against N Proteins of SNV/ANDV		Against PUUV-Vra N Protein Segment (aa 1-120)			
										4H3 ^a^	7G2 ^a^	2C6 ^b^	5C5 ^b^	5E11 ^b^	7A5 ^b^
DOBV-Slo	+	+	+	+	+	−	−	−	−	− *	− *	− *	− *	+ *	− *
DOBV-Slk	+	+	+	+	+	−	−	−	−	−	−	−	−	+	−
DOBV-Saa	+	+	+	+	+/−	−	−	−	−	−	−	−	−	+	−
HTNV	+	+	+	+	+	−	−	−	−	− *	+ *	− *	− *	− *	− *
SEOV	+	+	+	+	+	−	−	−	−	− *	+ *	− *	+ *	+ *	− *
THAIV	+	+	+	+	+	−	−	−	−	−	+	−	−	+	−
PUUV-Bawa	+	−	−	−	+	+	+	−	−	−	+	−	+	+	+
PUUV-Kaz	+/−	−	−	−	+/−	+	+	−	−	− *	+ *	− *	+ *	+ *	+ *
PUUV-Sot	+	−	−	−	+	+	+	−	−	− *	+ *	− *	+ *	+ *	+ *
PUUV-Vra	−	−	−	−	−	+	+	−	−	− *	+ *	+ *	+ *	+ *	+ *
TULV	−	−	+	−	+	−	−	−	−	− *	+ *	− *	+ *	+ *	+ *
PHV	+	−	+	−	+	−	−	−	−	−	+	−	+	+	+
SNV	+	−	+	−	+	−	−	−	−	+ *	+ *	− *	+ *	+ *	+ *
ANDV	+	+	+	+	+	−	−	−	−	+ *	+ *	− *	+ *	+ *	+ *
TPMV	−	−	−	−	−	−	−	+	+	−	−	−	−	−	−
SBV	−	−	−	−	−	−	−	−	−	−	−	−	−	−	−
RVFV	−	−	−	−	−	−	−	−	−	−	−	−	−	−	−
TSWV	−	−	−	−	−	−	−	−	−	−	−	−	−	−	−

*Dobrava-Belgrade orthohantavirus,* genotype Dobrava, strain Slovenia (DOBV-Slo), genotype Kurkino, strain Slovakia (DOBV-Slk) and genotype Saaremaa (DOBV-Saa); *Hantaan orthohantavirus* (HTNV); *Seoul orthohantavirus* (SEOV); *Thailand orthohantavirus* (THAIV); *Puumala orthohantavirus* strains Bavaria (PUUV-Bawa), Kazan (PUUV-Kaz), Sotkamo (PUUV-Sot), Vranica/Hällnäs (PUUV-Vra); *Tula orthohantavirus* (TULV); *Prospect Hill orthohantavirus* (PHV); *Sin Nombre orthohantavirus* (SNV); *Andes orthohantavirus* (ANDV); *Thottapalayam thottimvirus* (TPMV); *Schmallenberg orthobunyavirus* (SBV); *Rift valley fever phlebovirus* (RVFV)*; Tomato spotted wilt tospovirus* (TSWV). Reactivity of the MAbs is shown as follows: −, negative; +, positive; +/−, weak reactivity. ^a^, ^b^—MAbs described in [47,49], respectively. *—data taken from [47].

**Table 4:** Reactivities of monoclonal antibodies with hantavirus-infected cells in IFA.

Hantaviruses Used in the Test	Reactivity of Hantavirus-Specific MAbs												
	Novel MAbs							Previously Generated MAbs					
	DOBV 2	DOBV 3-2	DOBV 4	DOBV F2-2	TULV 1	PUUV 1	PUUV 10-3	Against N Proteins of SNV/ANDV		Against PUUV-Vra N Protein Segment (aa 1-120)			
								4H3 ^a^	7G2 ^a^	2C6 ^b^	5C5 ^b^	5E11 ^b^	7A5 ^b^
DOBV-Slo	++	+	++	+	++	−	−	−	−	−	−	++ (+)	−
DOBV-Saa	++	+	++	+	+	−	−	−	−	−	−	++ (+)	−
HTNV	+++	+	+++	+	+++	−	−	−	++ (+)	−	−	−	−
SEOV	++	+	++	+	+++	−	−	−	+	−	− (+)	++ (+++)	−
PUUV-Kaz	−	−	−	−	++	+	+	−	+	+++	+++	+++	+ (+++)
SNV	+++	−	++	−	+++	−	−	+++	++	−	+++ (++)	+++	++
ANDV	+++	++	++	++	+++	−	−	+++	++ (−)	−	+++ (+)	+++ (++)	+ (−)

*Dobrava-Belgrade orthohantavirus*, genotype Dobrava, strain Slovenia (DOBV-Slo); genotype Saaremaa (DOBV-Saa); *Hantaan orthohantavirus* (HTNV); *Seoul orthohantavirus* (SEOV); *Puumala orthohantavirus* strain Kazan (PUUV-Kaz); *Sin Nombre orthohantavirus* (SNV); *Andes orthohantavirus* (ANDV). The symbols “+”, “++” and “+++” refer to the intensity of the fluorescence signal; negative reactions are indicated as “−”. ^a^, ^b^—MAbs described by [47,49], respectively. If the current reactivity differs from that described previously in [47], it is indicated before the brackets.

## Data Availability

All data are provided within the manuscript and its Supplementary Materials.

## Supplementary Materials

The following supporting information can be downloaded at: https://www.mdpi.com/article/10.3390/v15020532/s1, Table S1: Oligonucleotides used in this work; Table S2: List of constructed plasmids for the synthesis of entire and truncated N proteins of *Dobrava-Belgrade virus* (DOBV), *Tula virus* (TULV) and *Thottapalayam virus* (TPMV) in *E. coli*; Figure S1: Epitope mapping of hantavirus-specific MAbs; Figure S2: Western blot analyses of *E. coli*-produced N protein derivatives of PUUV strain Vranica/Hällnäs with (a) anti-His MAb, (b) MAb PUUV 1 and (c) MAb PUUV 10-3 cell culture supernatants; Figure S3: Reactivities of DOBV-, TULV-, TPMV- and PUUV-specific MAbs (a) and of control antibodies (b) in Western blot assays with hantavirus N proteins and N proteins of other bunyaviruses.
