# Supplementary material for: Characterization of a Panel of Cross-Reactive Hantavirus Nucleocapsid Protein-Specific Monoclonal Antibodies
Source: Viruses. 2023 Feb 14;15(2):532. doi: 10.3390/v15020532 (PMC9958643; doi:10.3390/v15020532)
Supplement: Supplementary file 1 [file viruses-15-00532-s001.zip › viruses-2147375-supplementary.pdf]

Table S1. Oligonucleotides used in this work.

Primer name	Primer sequence 5' to 3'	Restriction endonuclease
DOBV_F	TGCTCGAGACA**ATG**AGTGACTTGACAGACATTC	XhoI
DOBV_R	AGCTCGAGAAGCTTAAGCGGCTCCTGATTAG	XhoI
DOBV_N120_R	AGCTCGAGTAGCCAGTCTGCAGTTTGCCCTG	XhoI
TULV_F	TGTCTAGAACA**ATG**AGCCAACTCAAAGAAATAC	XbaI
TULV_R	AGTCTAGA**TTA**GATTTTTAGCGGTTCCTG	XbaI
TULV_N120_R	AGTCTAGA**TTA**AAACCAATCTGCTGTCTGTC	XbaI
TPMV_F	TGTCTAGAACA**ATG**ACTCAAGGGAAAATGAC	XbaI
TPMV_R	AGTCTAGA**TTA**CAGTTTAATAGGCTCCTGACTTG	XbaI
TPMV_N120_R	AGTCTAGA**TTA**TTCTAGGCTGAGTGGATTGAG	XbaI
TSWV_F	TGTCTAGAACA**ATG**TCTAAGGTTAAGCTCACTAAG	XbaI
TSWV_R	AGTCTAGA**TTA**AGCAAGTTCTGCGAGTTTTG	XbaI

XhoI and XbaI restriction endonuclease recognition sites are underlined; start and termination codons are shown in bold. Primers were synthesized by Metabion International AG (Planegg, Germany) and Invitrogen (Glasgow, UK).

Table S2. List of constructed plasmids for the synthesis of entire and truncated N proteins of *Dobrava-Belgrade virus* (DOBV), *Tula virus* (TULV) and *Thottapalayam virus* (TPMV) in *E. coli*.

Primers used for cloning	Plasmid used for the construction	Constructed plasmids	Synthesized protein
DOBV_F, DOBV_R	pET28a$^+$	pET28_DOBV_N	DOBV N
DOBV_F, DOBV_N120_R		pET28_DOBV_N120	DOBV N120
TULV_F, TULV_R		pET28_TULV_N	TULV N
TULV_F, TULV_N120_R		pET28_TULV_N120	TULV N120
TPMV_F, TPMV_R		pET28_TPMV_N	TPMV N
TPMV_F, TPMV_N120_R		pET28_TPMV_N120	TPMV N120

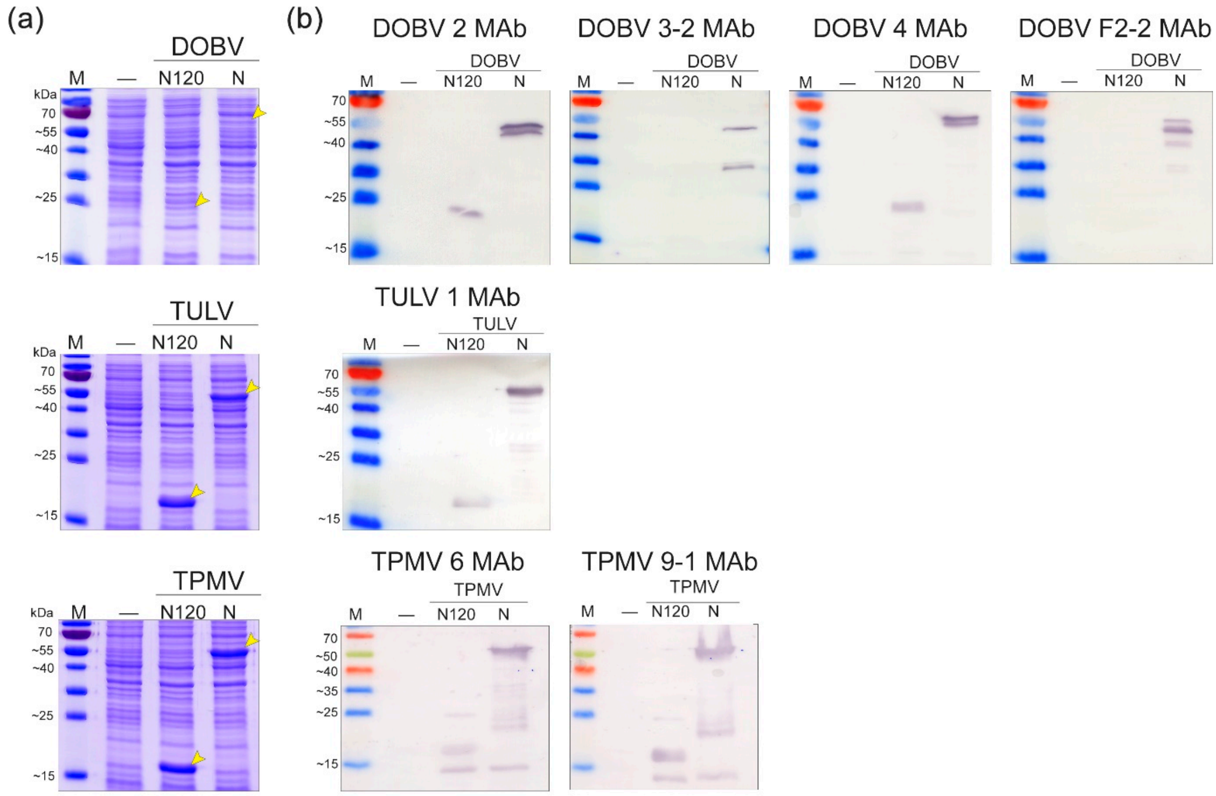

Figure S1. Epitope mapping of hantavirus-specific MAbs. **(a)** SDS-PAGE analysis of *E. coli* cell lysates expressing recombinant N proteins of DOBV, TULV and TPMV. "-" shows the lysate of bacteria that do not express a recombinant protein which was used as a negative control; "N" refers to the full-length hantavirus N proteins; "N120" refers to the truncated 120 aa-long proteins. Yellow arrows show bacteria-expressed truncated N120 and full-length proteins in cell lysate samples. **(b)** Reactivity patterns of hantavirus-specific MAbs with full-length and truncated N proteins of DOBV, TULV and TPMV in Western blot assay.

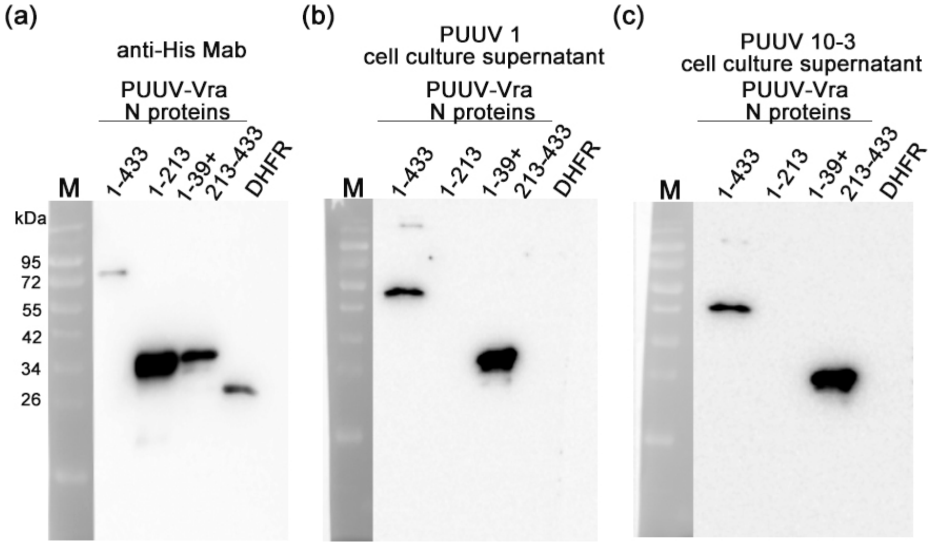

Figure S2. Western blot analyses of *E. coli*-produced N protein derivatives of PUUV strain Vranica/Hällnäs with **(a)** anti-His MAb, **(b)** MAb PUUV 1 and **(c)** MAb PUUV 10-3 cell culture supernatants. The numbers above each row indicate full-length (aa 1-433) and truncated N proteins (aa 1-213, aa 1-39+213-433) of *Puumala orthohantavirus*, strain Vranica/Hällnäs (PUUV-Vra). Mouse dihydrofolate reductase (DHFR) expressed in the same pQE-vector-based *E. coli* system and purified by the same affinity chromatography was used as negative control.

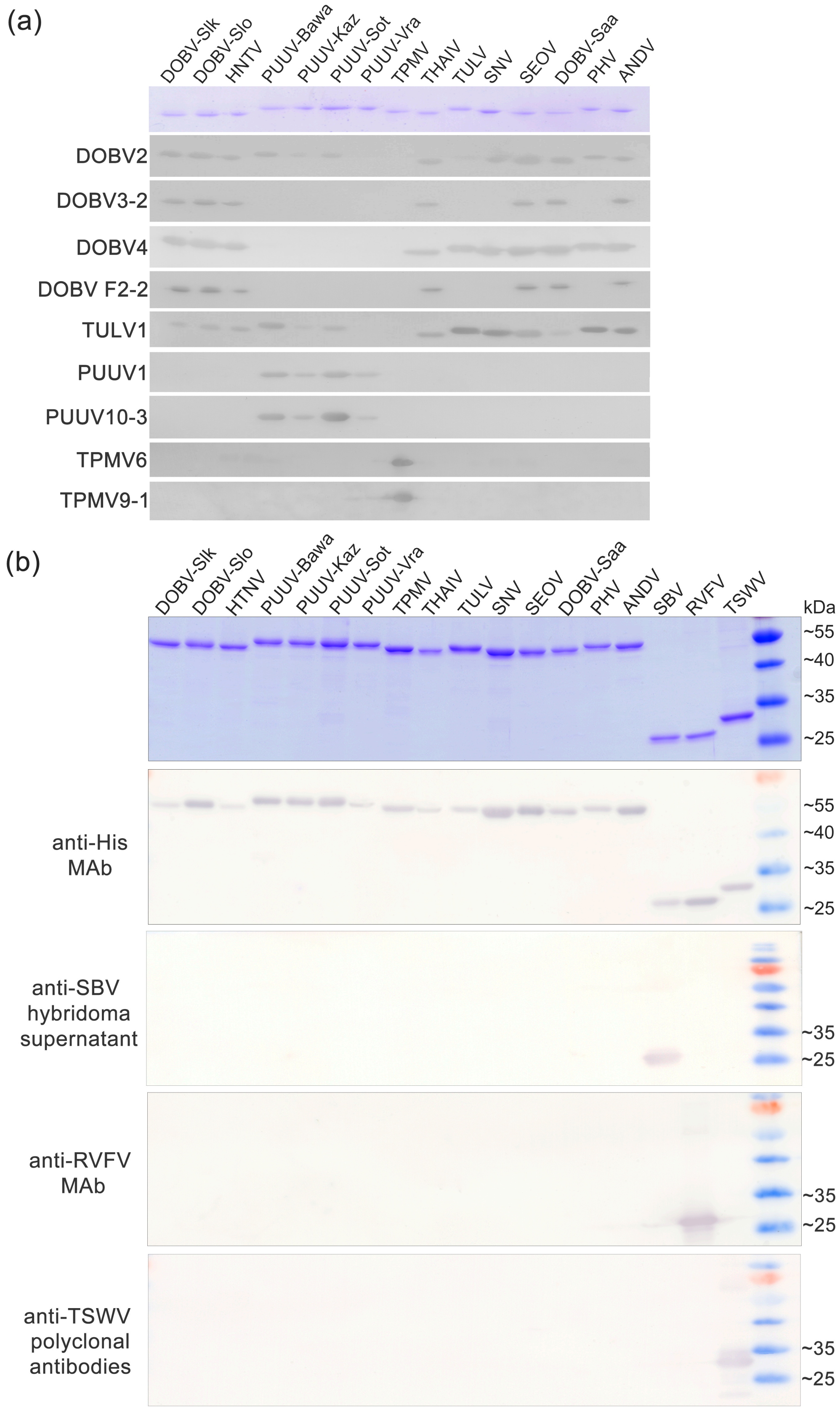

Figure S3. Reactivities of DOBV-, TULV-, TPMV- and PUUV-specific MAbs **(a)** and of control antibodies **(b)** in Western blot assays with hantavirus N proteins and N proteins of other bunyaviruses. A part of SDS-PAGE with fractionated hantavirus N proteins is shown in the first row of panels a and b (in blue color). The rows shown below demonstrate the reactivity of hantavirus-specific MAbs (a) and control antibodies raised against His-tag, SBV, RVFV or TSWV (b). The antibodies used are specified on the left of each row. *Dobrava-Belgrade*

orthohantavirus, genotype Kurkino, strain Slovakia (DOBV-Slk), genotype Dobrava, strain Slovenia (DOBV-Slo); *Hantaan orthohantavirus* (HTNV); *Puumala orthohantavirus*, strains Bavaria (PUUV-Bawa), Kazan (PUUV-Kaz), Sotkamo (PUUV-Sot), Vranica/Hällnäs (PUUV-Vra); *Thottapalayam thottimvirus* (TPMV); *Thailand orthohantavirus* (THAIV); *Tula orthohantavirus* (TULV); *Sin Nombre orthohantavirus* (SNV); *Seoul orthohantavirus* (SEOV); *Dobrava-Belgrade orthohantavirus*, genotype Saaremaa (DOBV-Saa); *Prospect Hill orthohantavirus* (PHV); *Andes orthohantavirus* (ANDV). Lower molecular weight having N proteins of *Schmallenberg orthobunyavirus* (SBV) (27 kDa), *Rift valley fever phlebovirus* (RVFV) (28.5 kDa) and *Tomato spotted wilt tospovirus* (TSWV) (29.7 kDa) used as negative controls are not shown in panel (a).
